# Fourier-Based Footfall Placement Variability in Parkinson's Disease

**DOI:** 10.1155/2019/2689609

**Published:** 2019-04-30

**Authors:** Sunghoon Shin, Bee-oh Lim, Michael J. Socie, Jacob J. Sosonff, Ki-Kwang Lee

**Affiliations:** ^1^School of Kinesiology, Yeungnam University, Gyeongsan, Republic of Korea; ^2^Department of Physical Education, Chung-Ang University, Seoul, Republic of Korea; ^3^Rehabilitation Institute of Chicago, Chicago, USA; ^4^Department of Kinesiology and Community Health, University of Illinois at Urbana-Champaign, USA; ^5^Department of Physical Education, Kookmin University, Seoul, Republic of Korea

## Abstract

The current investigation examined whether Parkinson's patients (PD) have greater Fourier-based footfall placement gait with the greatest mobility dysfunction variability (FPV) than the age and gender matched control group and that variability would be the greatest in the PD participants with the greatest mobility dysfunction indexed Hoehn/Yahr scale. 35 persons undergoing PD and 30 age-matched controls participated in this investigation. Participants repeated two trials' normal walking and average and variability parameters of gait were measured using a 3.66 m electronic walkway. FPV was quantified as a change in the center of pressure during gait. Persons with PD were divided into two groups based on Hoehn/Yahr scale. Overall, persons with PD had smaller average performance indexed by mean and greater gait variability than controls as indexed by CV and Fourier-based variability (p's<0.05). Moreover, PD with higher mobility dysfunction had not only greater variability in traditional parameters but also greater Fourier-based variability than nonfallers with MS (p<.001) with higher effect size (*η*^2^=0.37 vs.0.18-0.29). These observations highlight the fact that footfall placement variability is related to mobility dysfunction in PD. Further study is necessary to determine contributing factors to an increased FPV and whether targeted interventions such as exercise can reduce FPV.

## 1. Introduction

Parkinson's disease (PD), a kind of neurodisorder, occurs to about 1% of the elderly around 60 [1]. It was well known that gait disturbances of PD patients due to loss or impairment of dopaminergic innervation of the basal ganglia within central nervous system are a common cause of freezing of gait, alteration of gait pattern or regularity, and larger gait variability [1–11].

Normally, gait variability, that is, fluctuations in gait, is common to every person. Even though traditionally this behavioral fluctuation was considered as random noise, newly suggested dynamic system perspectives on movement variability have led to examinations of gait variability as a meaningful clinical scale, which may predict functional decline in daily movement [12–14]. Recent researches verified that gait variability contains complementary information about both function of locomotion and changes in walking due to aging and neuromuscular disease [1, 11, 15].

Gait is influenced by multiple interacting subsystems, (i.e., central nervous, musculoskeletal, and cardiovascular system) [16]. Organization of variability is challenged by functional decline in the operation of subsystems from healthy aging, degenerative, or chronic disease [16–18]. It was reported that gait variability stems from at least one of problematic limitations in the combined processes [1, 11]. There is evidence of increased gait variability in individuals with advanced age [19–23], Parkinson's disease [5, 24, 25], dementia [26], multiple sclerosis [27], diabetes mellitus [28], and hemodialysis [29, 30].

Gait variability, defined as temporal or spatial fluctuation of gait, is a valuable indicator of motor function [31]. Various studies have shown relationships between variability of different gait parameters (e.g., step width, step length, and double-support time) and other aspects of function such as walking speed, central nervous system impairment, or fall risk [19, 22, 32–34]. As such, the literature demonstrates that there are many possible variability parameters to choose from when analyzing gait and that there is no gold standard metric of gait variability.

Common metrics of gait variability are the standard deviation (SD) and coefficient of variation (CV). However, these distributional measures do not take into account any time-dependent aspects of fluctuations within the time series. Recently, a novel metric of spatial variability of footfall patterns based on Fourier analysis was introduced [16]. This method of quantifying foot placement variability may be advantageous compared with the traditional distributional variability parameters such as the SD or CV of step length or width in that it is useful for assessing spatial footfall placement variability in two-dimensional coordinates (i.e., anterior and posterior [AP] and mediolateral [ML] directions simultaneously) with harmonic numbers [16]. Moreover, it can be easily used in a relatively short time series and can be measured using a plantar pressure mat system that has been validated in previous studies [8, 35–37]. Given that accuracy of measurement and ease of use are the conditions for the gold standard measuring device, the aforementioned method can be considered a candidate gold standard analysis method for gait variability [38]. However, this Fourier-series-based metric was only used in the study of individuals with multiple sclerosis. Given the novelty of the variability measure in persons with MS, it is worthwhile to determine if this metric is sensitive to gait impairment in other pathologies including Parkinson's disease.

The purpose of this investigation was (1) to quantify gait variability in individuals with PD and controls using distributional metrics and a novel metric based on Fourier series and (2) to examine the association between gait variability and disease level in PD. We hypothesized that the PD group would have greater gait variability than the age and gender matched control group and that Fourier-based gait variability would be the greatest in the PD participants with the greatest motor impairment.

## 2. Materials and Methods

All procedures during this investigation were approved by an Institutional Review Board. All participants signed written informed consent.

### 2.1. Participants

Inclusion criteria for participants with PD required a neurologist-confirmed diagnosis. Individuals with PD, who were unable to walk without the support of other people, were excluded from this study. Therefore, two participants who used canes were included because they could walk independently. Inclusion criteria for healthy controls required no gait impairment, no history of falling in the last 12 month, and no chronic disease (e.g., diabetes mellitus) or neuromuscular or cardiovascular disease or dysfunction.

A total of 65 participants were recruited for this experiment. The control group consisted of thirty age-matched healthy older adults (22 females, 8 males, average age = 66.06 yrs, SD = 3.61 yrs). The control group was recruited through local advertisements and oral communication. After evaluation by a neurologist, the participants with PD were divided into two groups. According to the Hoehn/Yahr scale, low-level PD (Hoehn and Yahr scale 1-2) consisted of twenty-two individuals (14 females, 8 males, average age = 65.36 yrs, SD = 8.59 yrs). High PD (Hoehn and Yahr scale 3-4) consisted of ten individuals (6 females, 4 males, average age = 62.20 yrs, SD = 7.80 yrs) [39]. The criteria between low-level PD (Hoehn and Yahr scale 1-2) and High PD (Hoehn and Yahr scale greater than 2) are whether there was balance impairment during Parkinson's disease progress. Demographic data on the participants are provided in Table 1.

### 2.2. Instrumentation

The GAITRite® system (CIR Systems Inc., Peekskill, NY), a plantar pressure mat system, was used to collect gait data. The total length of the walkway was 4.88 m with pressure sensors covering an active area of 3.66 m long and 0.61 m wide. The mat was set up on a level surface for testing. The GAITRite® system's reliability and validity have been established [8, 35–37].

### 2.3. Experimental Procedure

The GAITRite® system was installed in the local hospital physical therapy room. Participants with PD were tested in their medicated state. Participants performed two comfortable paced walks along a 22 m walkway. Participants started walking 8 m before they stepped onto the GAITRite® pressure mat and finished 8 m beyond. All walking trials were performed barefoot.

### 2.4. Data Reduction

The temporal-spatial and pressure distribution variables were calculated by the GAITRite software (version 3.2b). The validated measures provided by the manufacturer of GAITRite, functional ambulation profile (FAP) score, walking speed (cm/s), step width (cm), step length (cm), and step time (s) were calculated for gait performance [37]

Gait variability was quantified with 2 metrics: the coefficient of variation (CV=100% SD/mean) and Fourier-based variability. CV of step time, step length, and step width were calculated.* Fourier-based footfall placement variability* (FPV) was calculated from the center of pressure data captured by the gait mat [16]. The procedure of calculation was as follows. First, the footfall patterns over the walkway measuring the coordinates of the center of pressure (COP) on the gait mat during a single walk were captured. Second, spatial coordinates of COP trajectories were used. In order to quantify FPV, the sequence of footfalls was fit with a Fourier series of sine and cosine waves using MATLAB (version R2018b, MathWorks, Inc., Natick, MA, USA), following the form

**Figure 1:**
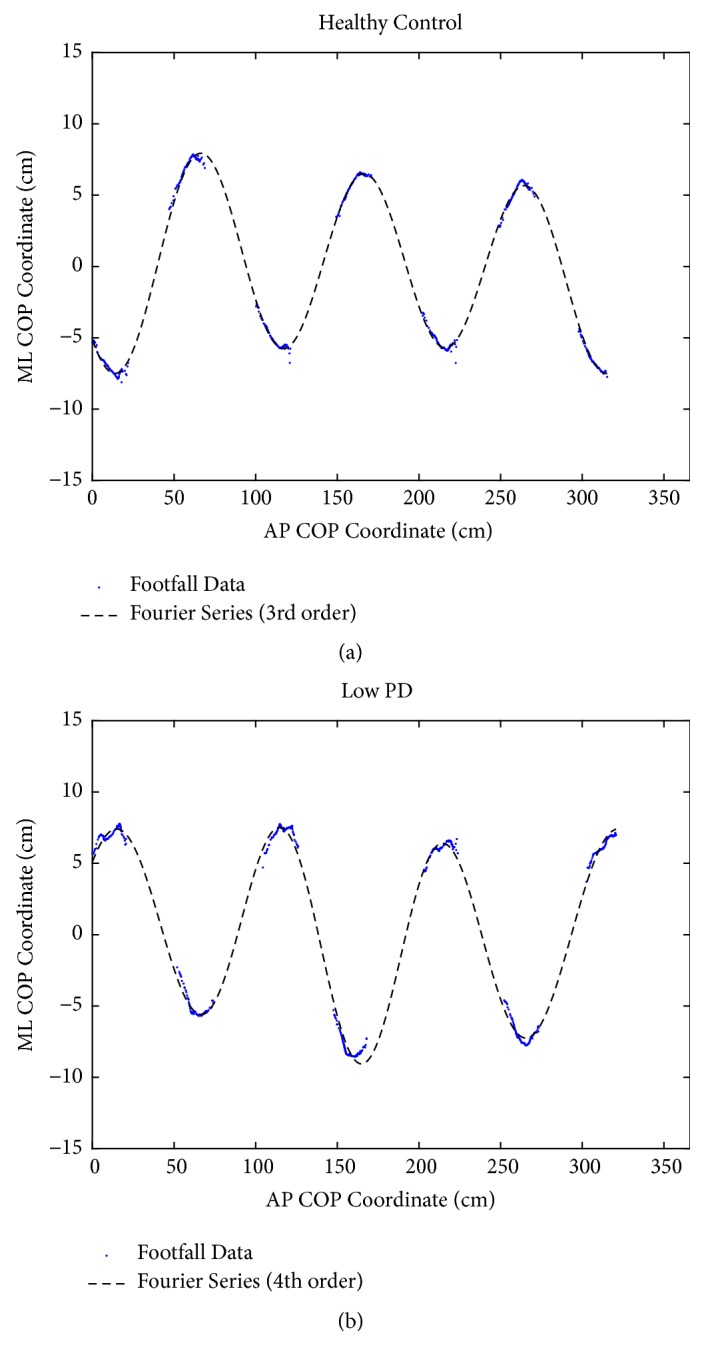
*Footfalls of a healthy and a low PD person.* (a) The COP coordinates of each footfall from a representative trial by a healthy control participant are shown. The footfall pattern was the best fit with a 3^rd^ order Fourier series (FPV=3). (b) The COP coordinates of each footfall from a representative trial by a person with low PD. The pattern was fit with a 4^th^ order Fourier series (FPV=4). The walking direction was left to right.

**Figure 2:**
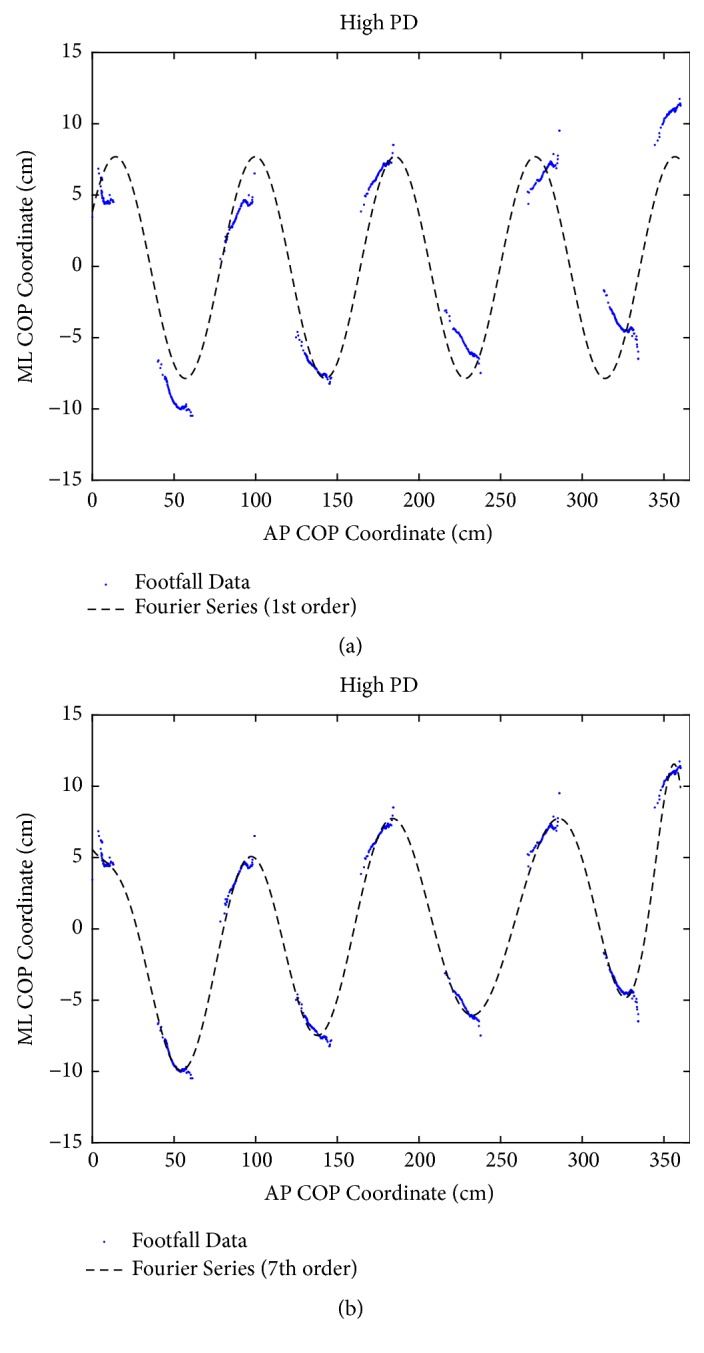
*Footfalls of a high PD person (1* ^*st*^ *vs. 7* ^*th*^ order Fourier series fit) (a) The COP coordinates of each footfall from a representative trial of a person with high PD are shown with a 1st order Fourier series fit. (b) A 7th order Fourier series (FPV=7), which was the best fit, is shown. The walking direction was left to right.

(1) y=∑i=1nAi×sin⁡π×i×ω×x+Bi×cos⁡π×i×ω×x,

where y is the COP coordinate in the ML plane, x is the COP coordinate in the AP plane, n is the series order, *ω* is frequency, and* A*_*i*_, and* B*_*i*_ are constants. More details are followed the previous study [16]. As Figures 1 and 2 showed, footfall sequence was fit with Fourier series of increasing order from one to eight until the error between the fitted curves. Error threshold value was five percent of footfall sequence.

### 2.5. Statistics

All statistical analyses were performed in SPSS version 20.0. Data were averaged from both walking trials for analysis. A one-way analysis of variance (ANOVA) was performed to determine group differences between the three groups (controls, low PD, and high PD). In addition, to separate the main effect of groups from walking speed, an analysis of covariance (ANCOVA) with groups as between-subject factors and walking speed as the covariate was conducted. A Tukey post hoc analysis was conducted for multiple comparisons between each group. *η*^2^was calculated to evaluate the effect size of the one-way ANOVA and ANCOVA [40]. For *η*^2^, .01, .06, and .14 were considered small, moderate, and large effects, respectively. Group differences in gender distribution were accessed with a Mann-Whitney U test. Spearman correlations were performed between Fourier-based variability and CV of step time, step length, and step width.

## 3. Results

Demography of subjects including age, gender, Hoehn/Yahr scale, and assistive device use is reported (Table 1). There were no significant differences in age, weight, height and sex distribution (P>.05). Only two participants in high Parkinson's disease patients used assistive devices.

There were group differences in walking velocity and average gait parameters (Table 2). The control group walked significantly faster than both PD groups (about 30 and 40 %, resp., p's*≤*0.001). Low PD walked 17 % faster than high PD (p*≤*0.001). The differences in walking speed between controls and PD groups coincided with decreased step length of PD patients (Table 2). However, there were no significant differences in step time among three groups (p's>.05). The overall effect size of three group differences of walking speed and average gait parameters was large *η*^2^ with magnitude ranging from 0.21 to 0.48.

Both PD groups demonstrated significantly greater Fourier-based variability and step time CV than the control group (p's<.001) (Table 3). There was no significant difference in step length CV between low PD and controls (p>.05). However, between PD groups, high HD group had greater step width CV, step length CV, step time CV, and Fourier-based variability than low PD group (p's<.001) (Table 3). The overall effect size of all group differences in gait variability was large, with *η*^2^magnitude ranging from 0.18 to 0.41. The result of the ANCOVA indicated that the effect of group was observed only on FPV (F_[2,61]_ = 7.44, p < .01, *η*^2^ = 0.20) and step time CV (F_[2,61]_ = 3.46 p < .05, *η*^2^ = 0.10) after controlling for the effect of walking speed.

In order to examine whether Fourier-based variability was correlated to traditional gait variability measures (i.e., coefficient of variation), Spearman rank-ordered correlations were conducted. Fourier-based variability was significantly correlated with step width CV ( = 0.527, *p* < .001), step length CV (*ρ* = 0.573, *p* < .001), and step time CV (*ρ* = 0.610, *p* < .001).

## 4. Discussion

The purpose of the current investigation was to determine the sensitivity of Fourier-based gait variability to distinguish between individuals with PD and compare Fourier-based variability to traditional spatiotemporal gait variability metrics in individuals with PD. Fourier-based gait variability was previously associated with disability status and fall history in individuals with multiple sclerosis [16]. It was hypothesized that persons with PD would have greater gait variability than healthy controls and the severity of PD (graded by Hoehn/Yahr scale) would be correlated with Fourier-based variability measure.

Overall there were two novel observations. First, PD patients had greater gait variability in both traditional measures (i.e., coefficient of variation) and Fourier-based gait variability compared to controls. Second, Fourier-based gait variability was significantly greater in individuals with more progression of PD than individuals with more mild PD. Even though the current sample size was relatively small, the results consistently support our hypothesis that gait impairment of persons with PD could be evaluated using Fourier-based gait variability with large effect size (*η*^2^ = 0.37). Moreover, this metric scale of high PD patients is larger than low PD patients. This suggests that Fourier-based variability is sensitive to both mobility dysfunction as indexed by Hoehn/Yahr scale [39] and variability of individual spatiotemporal gait parameters. As a difference in walking speed was found among the groups, whether walking speed contributed to the gait variability difference was of interest. The main effects of mobility dysfunction as indexed by Hoehn/Yahr scale are independent of gait speed for the Fourier-based variability, with a large effect size (*η*^2^ = 0.20). The results indicated that the Fourier-based gait variability is an estimator independent of walking speed.

Fourier-based footfall placement variability was related with all traditional gait variability parameters. This finding is in contrast to previous work utilizing this metric which reported that Fourier-based variability was related to step length and step time variability but not step width variability [16]. A possibility is that the subject of current study relatively is older than the previous study (about 52.5 yrs vs. 66.7 yrs) and another potential explanation for this discrepancy is that the Fourier-based footfall placement variability is sensitive enough to reflect different spatiotemporal gait coordination patterns across impairment groups (i.e., MS and Parkinson's diseases patients). Our results showed relatively about two times larger FPV values of PD patients (about 9.5 to 18.8) than MS patients (about 7.0). It may imply that the severity of gait impairment in PD patients has more than MS and could differentiate the gait patterns. Gait variability of MS results from increased spasticity, fatigue, and declined muscle strength [16]. Rather gait impairment of PD came from combined results from basal ganglia dysfunction and certain episodic symptoms [1]. The modified mechanisms could be the different combined results from diverse systems.

It has been suggested that Fourier-based gait variability may present unique information about gait patterns. Fourier-based gait variability incorporates spatial variability in both AP and ML directions simultaneously with larger effect size than traditional parameters (0.37 vs. 0.18 to 0.29). It was reported that sensorimotor (e.g., vestibular) impairment of the persons with PD was limited to walk regularly [1, 5]. In this meaning, Fourier-based variability could be still useful index to evaluate gait performance of persons with gait impairments (e.g., stroke, spinal cord injury, and arthritis patients) influenced by motor coordination in both AP and ML directions. A different aspect of gait variability measures could be different constructs of complicate locomotor mechanism [39].

Although the novel observations highlighted the use of Fourier-based variability to evaluate gait variability of the persons with PD, there were several limitations to the current study. First, even though participants were examined at the peak dose effect from their medication, there could be a potential distortion of the results from the medication. Second, gait measures were calculated from a relatively small number of steps, limited by the length of the gait mat and the number of trials that could safely be performed with the participants. Finally, the participants' physical activity level could be considered with other scales' indexing physical activity to give a complete picture of their physical capabilities.

## 5. Conclusions

In summary, gait variability, including Fourier-based variability, is increased in individuals with PD compared with controls. Furthermore, the Fourier-based variability of persons with low PD was statistically distinct from that of high PD. This finding demonstrates a relationship between disease progression and gait variability in individuals with PD.

## Figures and Tables

**Table 1:** Participant demographics.

	Controls (N=35)	Low PD (N=20)	High PD (N=10)	F (2, 61), P
Age (years)	67.80±6.29	67.25±6.55	62.10±7.69	1.345, 0.268

Height (cm)	157.88±6.37	158.50±6.62	161.20±10.18	1.111,0.336

Weight (Kg)	57.95±7.25	60.20±9.77	58.20±10.95	0.983,0.380

Gender	13M/22F	8M/12F	3M/7F	-

Hoehn/Yahr scale	None	1-2	3-4	-

Assistive device (cane/walker)	0/0	0/0	2/0	-

Low-level PD is the Hoehn and Yahr scale 1-2. High PD is the Hoehn and Yahr scale greater than 2.

**Table 2:** Average gait parameters of controls and low and high Parkinson's disease (mean±SD).

	Controls	Low PD	High PD	F (2, 62), P	*η* ^2^
FAP	91.40±9.07	83.30±16.26	73.7±15.51, ^c^	8.309, <.001	0.21

Walking speed (cm/s)	125.17±23.36	89.81±24.59^a^	74.94±25.25 ^b^	24.018, <.001	0.43

Step width (cm)	64.83±9.63	49.88±10.75 ^a^	41.87±9.47 ^b,c^	27.405,<.001	0.47

Step length (cm)	63.94±9.89	48.08±11.36 ^a^	38.92±10.59 ^b,c^	28.917,<.001	0.48

Step time (s)	0.51±0.08	0.54±0.07	0.53±0.09	0.573, 0.567	0.02

a, b, and c denote specific group differences from post hoc analysis. ^a^p<.05 difference between low PD and controls. ^b^p<.05 difference between high PD and controls. ^c^p<.05 difference between PD groups. FAP means functional ambulation profile score in GAITRite.

**Table 3:** Gait variability parameters (CV) of controls and low and high Parkinson's disease (mean±SD).

	Controls	Low PD	High PD	F (2, 62), P	*η* ^2^
FPV	2.86±1.06	3.87±1.67 ^a^	6.10±1.84 ^b,c^	21.159,<.001	0.41

Step width CV (%)	3.94±1.80	6.38±4.21 ^a^	7.76±4.97 ^b,c^	6.872,<.01	0.18

Step length CV (%)	4.06±2.00	7.29±5.44	10.73±10.08 ^b,c^	7.422,<.001	0.19

Step time CV (%)	3.40±1.50	5.09±2.65 ^a^	8.57±5.78 ^b,c^	12.886,<.001	0.29

a, b, and c denote specific group differences from post hoc analysis. ^a^p<.05 difference between low PD and controls. ^b^p<.05 difference between high PD and controls. ^c^p<.05 difference between PD groups. FPV means Fourier-based footfall placement variability. CV means coefficient of variance.

## Data Availability

The data used to support the findings of this study are included within the article.
